# Transcription Profile Analysis Reveals That Zygotic Division Results in Uneven Distribution of Specific Transcripts in Apical/Basal Cells of Tobacco

**DOI:** 10.1371/journal.pone.0015971

**Published:** 2011-01-07

**Authors:** Ligang Ma, Haiping Xin, Lianghuan Qu, Jing Zhao, Libo Yang, Peng Zhao, Mengxiang Sun

**Affiliations:** Key Laboratory of the Ministry of Education for Plant Developmental Biology, College of Life Science, Wuhan University, Wuhan, China; Temasek Life Sciences Laboratory, Singapore

## Abstract

**Background:**

Asymmetric zygotic division in higher plants results in the formation of an apical cell and a basal cell. These two embryonic cells possess distinct morphologies and cell developmental fates. It has been proposed that unevenly distributed cell fate determinants and/or distinct cell transcript profiles may be the underlying reason for their distinct fates. However, neither of these hypotheses has convincing support due to technical limitations.

**Methodology/Principal Findings:**

Using laser-controlled microdissection, we isolated apical and basal cells and constructed cell type-specific cDNA libraries. Transcript profile analysis revealed difference in transcript composition. PCR and qPCR analysis confirmed that transcripts of selected embryogenesis-related genes were cell-type preferentially distributed. Some of the transcripts that existed in zygotes were found distinctly existed in apical or basal cells. The cell type specific *de novo* transcription was also found after zygotic cell division.

**Conclusions/Significance:**

Thus, we found that the transcript diversity occurs between apical and basal cells. Asymmetric zygotic division results in the uneven distribution of some embryogenesis related transcripts in the two-celled proembryos, suggesting that a differential distribution of some specific transcripts in the apical or basal cells may involve in guiding the two cell types to different developmental destinies.

## Introduction

In some angiosperms, including *Arabidopsis* and tobacco, the first zygotic cell division is transverse and asymmetric, and results in a two-celled proembryo consisting of an apical cell and a basal cell, which differ in both their morphology and destiny [1]–[3]. The smaller apical cell develops into the embryo proper, while the larger basal cell develops into a suspensor or joins the embryonic root formation [4].

It has been a mystery for many years how apical and basal cells, which are descended from the same mother cell, show distinct divisional patterns and cell fates. To explain their distinct cell fates, it has been proposed that asymmetric divisions generate daughter cells containing different developmental determinants [5] or that the different developmental pathways of the cells occur due to different positional cues [6]. However, neither of these proposals has been demonstrated.

Cytoplasmic determinants play a predominant role in cell fate determination [7]. Researchers have identified several genes that are expressed differently in progeny after zygote division. In *Arabidopsis*, *MERISTEM LAYER 1* (*AtML1*) encodes a homeobox gene, and its expression has been confirmed only in the apical cell of two-celled proembryos [8]. In *Phaseolus coccineus*, the transcripts of two genes, G564 and G541, accumulate shortly after fertilization and are present within the two embryonic basal cells at the four-cell stage [5]. It was recently [9] reported that the transcription factors *WUSCHEL HOMEOBOX2* (*WOX2*) and *WOX8* are expressed specifically in the apical and basal cells of the *Arabidopsis* two-celled proembryo. These data suggest that the two zygotic daughter cells may assume different transcriptional profiles, although no evidence has been presented. Thus, a direct apical and basal cell transcriptional profile analysis would be useful in unraveling this mystery.

Because the zygote and early embryo are deeply embedded in the ovular tissue and are therefore not easy to access, it has been difficult to identify the transcriptome and detect dynamic changes in gene expression. In the last 20 years, techniques have been established to isolate gametes and early-stage embryos from a number of flowering plant species [10], and these specific cells have become available for direct use in large-scale analyses such as cDNA library construction and microarray analysis. Using *in vitro*-fertilized zygote culture, apical and basal cells have been isolated from maize, and apical and basal cell-specific genes have been identified using RAPD primers [11]. Expression pattern analysis revealed that the genes are upregulated in the apical or basal cell in the early zygote, suggesting that the transcripts are portioned in their respective cells after zygote division, or that the transcripts are rapidly degraded in one of the daughter cells after zygotic cell division [11]. However, until now, data on the apical and basal cell transcriptome have been lacking.

Since it is still very difficult to isolate zygote and two-celled proembryos from *Arabidopsis* and we have well established relevant techniques in tobacco [3], [12], in this report, we present a comparison of the transcript profiles between the two zygotic daughter cells of tobacco, and we compare the two cell types with the zygote. We also address two questions: 1) Do apical and basal cell possess distinct transcript profiles that may be responsible for their distinct cell fates? and 2) Can zygotic transcripts be portioned into the different daughter cells, which may involve in cell fate regulation. Our data suggest that the transcript diversity occurs between apical and basal cells. Asymmetric zygotic division result in uneven distribution of some specific transcripts in two daughter cells, thereby triggering their distinct developmental pathways.

## Results

### Isolation of apical and basal cells

**Figure 1:**
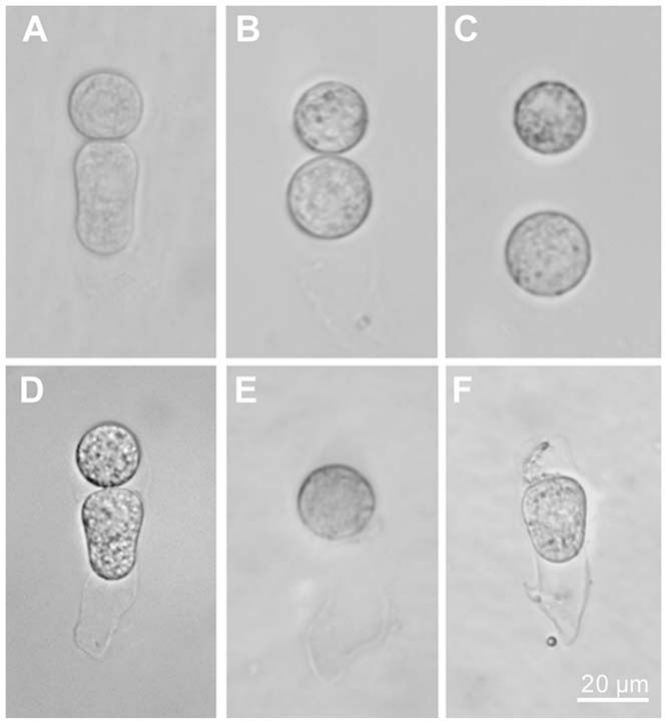
Isolation of two-celled proembryo, apical, and basal cells. (A) Isolated two-celled proembryo; (B) Apical and basal cell protoplasts; (C) Separation of apical and basal cell protoplasts; (D) Two-celled proembryo for laser ablation; (E) Apical cell ablation of basal cell; (F) Basal cell after ablation of apical cell. Bar = 20 µm.

Because the two-celled embryos of tobacco are deeply embedded in the ovules and are difficult to approach, viable two-celled proembryos must be isolated from ovules by enzymatic maceration combined with grinding [13]. We tested two different techniques to isolate apical and basal cells. When isolated two-celled proembryos were placed in an enzymatic solution for an extended duration, the apical and basal cells could be completely separated and became protoplasts (Figure 1A–C). We also used a laser microdissection device (LMD) to quickly ablate the apical and basal cells, respectively, and, after a brief washing procedure, individual apical or basal cells were collected (Figure 1D–F). As long-term enzymatic treatment may weaken cell viability and promote stress-induced gene expression, which might greatly alter the expression profile of the cells, we finally chose LMD to isolate the cells. During enzymatic treatment of the ovules to isolate two-celled proembryos, two transcriptional inhibitors, actinomycin D (50 mg/L) and cordycepin (100 mg/L) were added to all solutions [12], [14] to suppress stress-induced gene expression. After laser ablation, individual apical or basal cells were collected manually and immediately washed to avoid possible contamination from the broken cells. A pure population of apical or basal cells was collected efficiently using this method and all the cells were viable (Figure S1).

### Library construction and expressed sequence tag (EST) cluster analysis

Seventy-two apical cells and 88 basal cells were used to construct two cell type-specific cDNA libraries. The titers of the unamplified apical and basal cell libraries were 2.5×10^6^ and 2×10^6^ pfu·ml^−1^, respectively. More than 99% of the clones had an insert, indicating that the libraries were of high quality. The insert size of the cDNA clones in the apical cell cDNA library ranged from 0.3 to 1.7 kb (centered around 0.6 kb), compared to 0.3 to 1.7 kb in the basal cell cDNA library (centered around 0.5 kb).

EST sequencing was performed from the 5′-end of randomly picked clones from the two primary libraries. A total of 2,772 ESTs from the apical cell cDNA library were obtained and assembled into 2,072 non-redundant clusters containing 1,750 singletons; 2,776 ESTs were assembled into 1,950 non-redundant clusters containing 1,532 singletons for the basal cell cDNA library. In total, 45.42% of the clusters from the apical cells and 43.85% of the clusters from the basal cells could be assigned putative functions. Some of the clusters (25.63% for the apical cells and 28.92% for the basal cells) showed no significant homology with National Center for Biotechnology Information (NCBI) databases. The remaining clusters (28.96% for the apical cells and 27.23% for the basal cells) displayed similarities without specific annotations.

**Figure 2:**
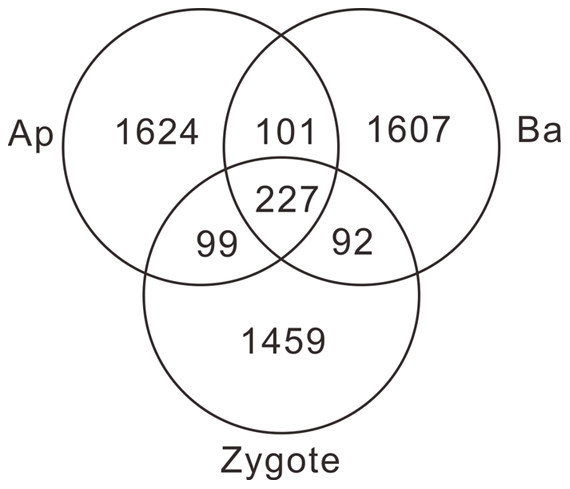
Apical and basal cell expressed sequence tag (EST) clusters compared to zygote EST clusters. EST cluster comparison between apical (Ap)/basal cell (Ba) ESTs and a zygote.

Our results showed that 16.0% of the apical cell EST clusters were present in basal cells and that 16.2% of the basal cell EST clusters were present in apical cells (Figure 2). Since this is EST-based and sampling analysis, the diversity of the transcript profiles between the two cell types needs to be further confirmed.

### Apical and basal cells share the majority of their most abundant transcripts

We further compared the transcript component between the two cell types. The most abundant clusters in apical and basal cells are shown in Tables 1 and 2, respectively. Several clusters containing ESTs related to ribosomal proteins, chaperones, ubiquitin, calmodulin, and histones appeared in both the apical and basal cells as abundant clusters, suggesting that apical and basal cells share the majority of their most abundant transcripts.

**Table 1:** Most abundant clusters in tobacco apical cells.

Cluster id	EST No.	AGI	Putative identity	e-value
**NtAc 1**	21	AT1G14980	chaperone	2.00E-30
**NtAc 2**	21	No hit		
**NtAc 3**	18	ATMG00030	NitaMp027	9.00E-36
**NtAc 4**	14	AT5G65360	histone H3	5.00E-71
**NtAc 5**	10	AT4G33865	40S ribosomal protein S29	3.00E-28
**NtAc 6**	9	AT3G46030	HTB11; DNA binding	1.00E-65
**NtAc 7**	9	AT3G12410	3′-5′ exonuclease/ nucleic acid binding	4.00E-58
**NtAc 8**	9	AT5G65360	histone H3	1.00E-70
**NtAc 9**	9	AT3G43810	calmodulin	9.00E-78
**NtAc 10**	8	AT5G02560	HTA12; DNA binding	2.00E-64
**NtAc 11**	8	AT2G09990	40S ribosomal protein S16	3.00E-70
**NtAc 12**	8	AT5G57290	60S acidic ribosomal protein P3	1.00E-41
**NtAc 13**	7	AT3G49010	structural constituent of ribosome	3.00E-93
**NtAc 14**	7	AT5G59910	HTB4; DNA binding	2.00E-64
**NtAc 15**	7	AT3G04400	structural constituent of ribosome	6.00E-76
**NtAc 16**	7	AT5G27670	H2A histone	5.00E-62
**NtAc 17**	7	AT5G27670	H2A histone	1.00E-61
**NtAc 18**	7	AT3G52590	ubiquitin extension protein	2.00E-69
**NtAc 19**	7	AT5G57290	60S acidic ribosomal protein P3	1.00E-40
**NtAc 20**	6	AT1G73230	BTF3 [Nicotiana benthamiana]	9.00E-65

**Table 2:** Most abundant clusters in tobacco basal cells.

Cluster id	EST No.	AGI	Putative identity	e-value
**Nt**B**c 1**	34	No hit		
**Nt**B**c 2**	34	ATMG00030	NitaMp027	8.00E-30
**Nt**B**c 3**	19	AT4G33865	40S ribosomal protein S29	6.00E-27
**Nt**B**c 4**	17	AT3G43810	calmodulin	2.00E-78
**Nt**B**c 5**	12	AT1G55020	lipoxygenase	2.00E-57
**Nt**B**c 6**	12	AT1G14980	chaperonin	9.00E-41
**Nt**B**c 7**	11	AT5G65360	histone H3	4.00E-71
**Nt**B**c 8**	10	AT4G39340	unknown protein	4.00E-28
**Nt**B**c 9**	9	AT5G59850	40S ribosomal protein S15A	2.00E-70
**Nt**B**c 10**	9	No hit		
**Nt**B**c 11**	8	AT3G04400	60S ribosomal protein L17	6.00E-76
**Nt**B**c 12**	8	AT1G55020	lipoxygenase	8.00E-64
**Nt**B**c 13**	7	AT3G52590	ubiquitin extension protein	4.00E-69
**Nt**B**c 14**	7	AT3G46030	HTB11; DNA binding	2.00E-65
**Nt**B**c 15**	7	No hit		
**Nt**B**c 16**	7	AT3G59540	60S ribosomal protein L38	1.00E-31
**Nt**B**c 17**	7	AT4G33865	40S ribosomal protein S29	3.00E-28
**Nt**B**c 18**	6	AT3G46030	HTB11; DNA binding	2E-65
**Nt**B**c 19**	6	AT1G26880	60S ribosomal protein L34 (RPL34A)	3.00E-59
**Nt**B**c 20**	6	AT5G59970	histone H4	1E-53

### Functional category analysis indicates strong similarity between apical and basal cells

**Figure 3:**
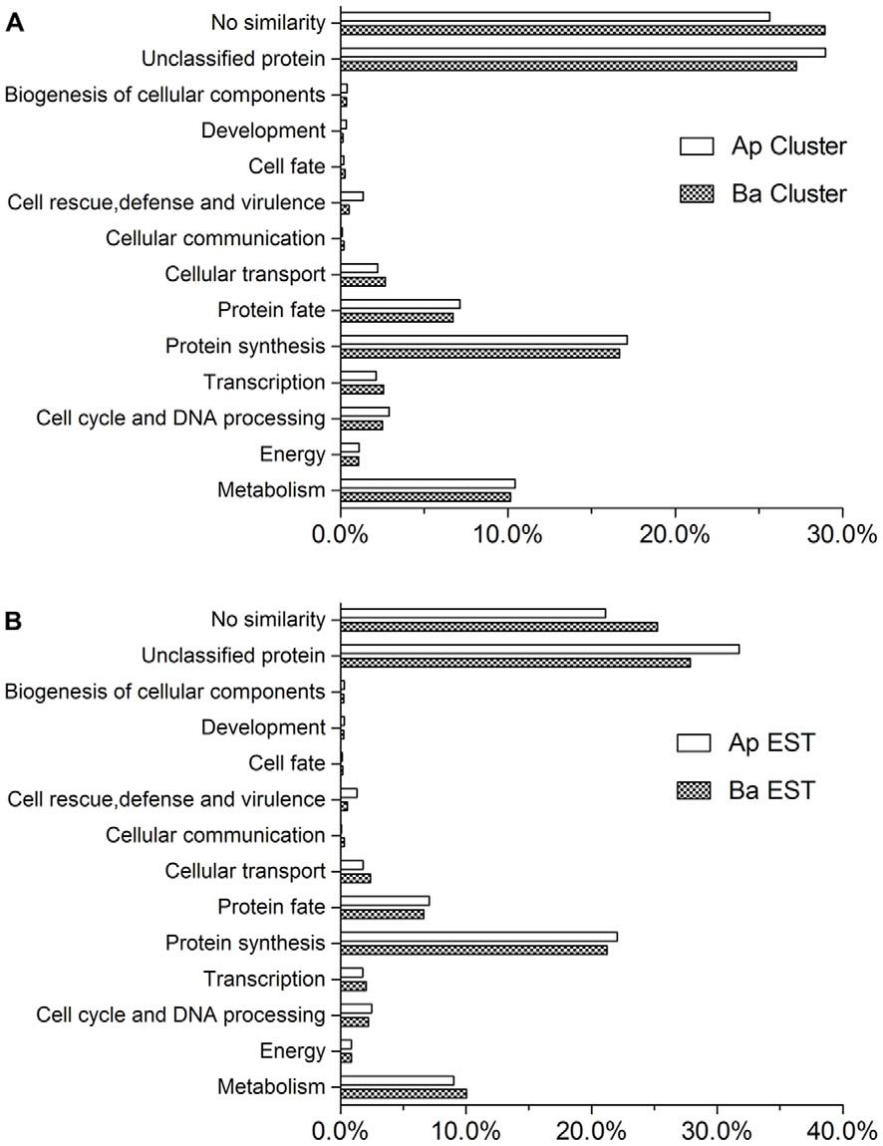
Functional cluster categories and expressed sequence tags from apical and basal cells. **A**: comparison of clusters between apical and basal cell. **B**: comparison of ESTs between apical and basal cell. Ap, apical cell; Ba, basal cell.

The clusters and ESTs matching-characterized proteins or proteins with putative functions were grouped according to functional categories (Figure 3). Similar to the results of our analysis of the most common clusters in apical and basal cells, the functional categories showed a high degree of similarity between the cell types. No distinct functional group of transcripts was found in either cell type. Because apical and basal cells undergo cell division to generate the embryo and suspensor, it is reasonable that most of the annotated clusters and ESTs were related to protein synthesis, metabolism, and DNA processing in both cells. The similar functional categories in apical and basal cells suggest that the cells inherit parallel groups of transcripts from zygote.

### Existence confirmation of clusters in apical and basal cells by RT-PCR

To further confirm the existence of the clusters from the two cDNA libraries, 34 clusters from the apical cell cDNA library and 37 clusters from the basal cell cDNA library were selected for RT-PCR. The clusters used for the analysis were involved in several biological processes (Table S1). These chosen clusters were estimated as numerous in one cell type; thus, the results will help evaluate whether particular clusters in a given cell type could be cell type-specific. The PCR parameters were optimized to identify ESTs that differed dramatically between apical and basal cells: 25 cycles for house-keeping genes and 35 cycles for candidate genes.

**Figure 4:**
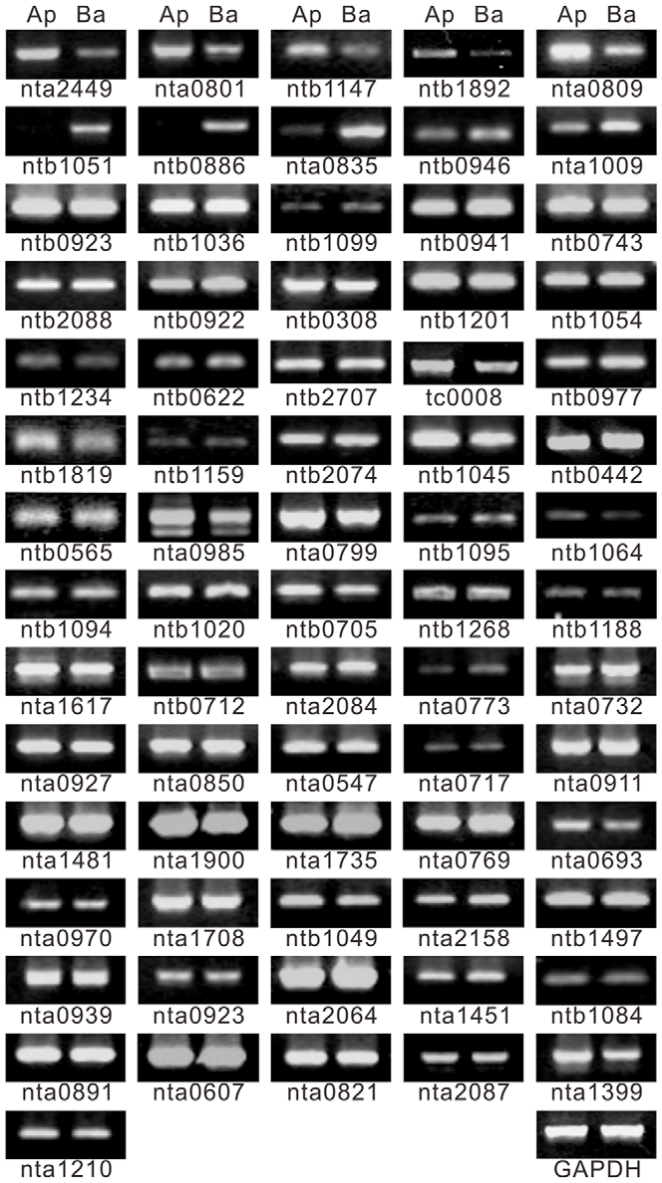
RT-PCR examination of transcripts in apical and basal cells. Of 71 expressed sequence tags (ESTs) examined by RT-PCR, only ntb1051 and ntb0886 could be confirmed exclusively in basal cells. The transcripts of five genes, nta0801, nat0809, ntb1147, ntb1892, and nta2449, were accumulated at a higher level in apical cells, whereas transcripts of three genes, nta0893, nta1009, and ntb0946, were accumulated more in basal cells.

Among the 34 clusters in the apical cell cDNA library (Figure 4), five showed slight reduction in basal cells, and none were reduced in apical cells. Among the 37 clusters from the basal cells, two in the basal cell cDNA library existed in basal cells and not in apical cells. Although the tested samples were limited, large-scale distinct transcript portioning could not be confirmed in the apical or basal cells during asymmetric zygotic division. But, some of transcripts were indeed specifically localized in one of the cell types.

To trace the fate of the transcripts identified from apical or basal cells in the subsequent development stages, hundreds of transcripts from the two cDNA libraries were examined in global-stage proembryos and heart-stage embryos. Among 126 transcripts detected (49 from apical cells, 77 from basal cells), seven were not found in global-stage embryos (two from apical cells, five from basal cells). Among 166 ESTs detected (49 from apical cells, 117 from basal cells), fourteen were not detected in heart-stage embryos (four from apical cells, ten from basal cells). The transcripts tested are listed in Table S1. These data suggest that most of the transcripts identified in the two-celled proembryos still existed at the subsequent developmental stages, indicating their persist role in embryo development.

### Real-time RT-PCR reveals preferential transcript accumulation in apical or basal cells

**Figure 5:**
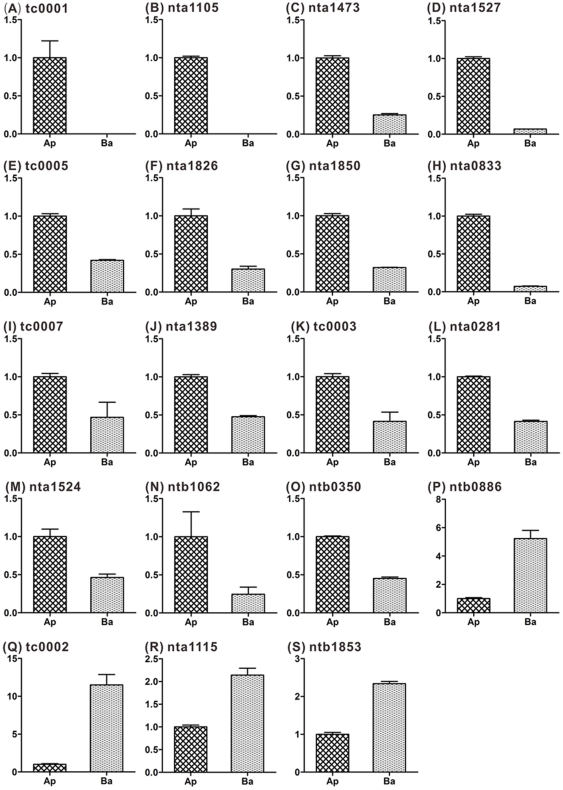
Transcript levels of selected genes in apical/basal cells as shown by real-time RT-PCR. (A, B): The expressed sequence tags (ESTs) showed specific distribution in apical cell. (C–O): ESTs showed a significantly higher level in apical vs. basal cells (≥2-fold difference). (P–S): ESTs showed a significantly higher level in basal vs. apical cells (≥2-fold difference). Ap, apical cell; Ba, basal cell; Zy, zygote. Expression level in apical cell was set to 1.

Our RT-PCR results indicated that most of the transcripts identified existed in both apical and basal cells. To detect the relative transcript level in the two cell types, 42 ESTs were examined by real-time RT-PCR. Among them, 19 ESTs showed a significant expression difference between apical and basal cells. Two ESTs (tc0001 and nta1105) were expressed exclusively in apical cells (Figure 5A, B) and other 13 ESTs(tc0003, tc0005, tc0007, nta0281, nta0833, nta1389, nta1473, nta1524, nta1527, nta1826, nta1850, ntb0350, ntb1062) showed significantly higher expression in apical cells(Figure 5C–O), whereas four ESTs (tc0002, nta1115, ntb0886, ntb1853) showed significantly higher expression in basal cells (Figure 5P–S). Three WOX-related ESTs (nta1115, ntb1853, and nta1527) had higher expression levels in apical and basal cells. nta1115 and ntb1853 are similar to WOX9 and were expressed at a higher level in basal than apical cells. nta1527 is similar to WOX2 and was expressed at a higher level in apical cells (Figure 5R, S). The three WOX-related ESTs examined were expressed in a similar pattern to WOX2/WOX9 in *Arabidopsis*. The expression levels of the other ESTs (Figure S2) were not significantly different between apical and basal cells. This data indicate that although most of the transcripts existed in both cells, some of embryogenesis-related transcripts are obviously polar distributed in two-celled proembryo.

### Most of the differentially accumulated transcripts in apical or basal cell were found in zygote

**Figure 6:**
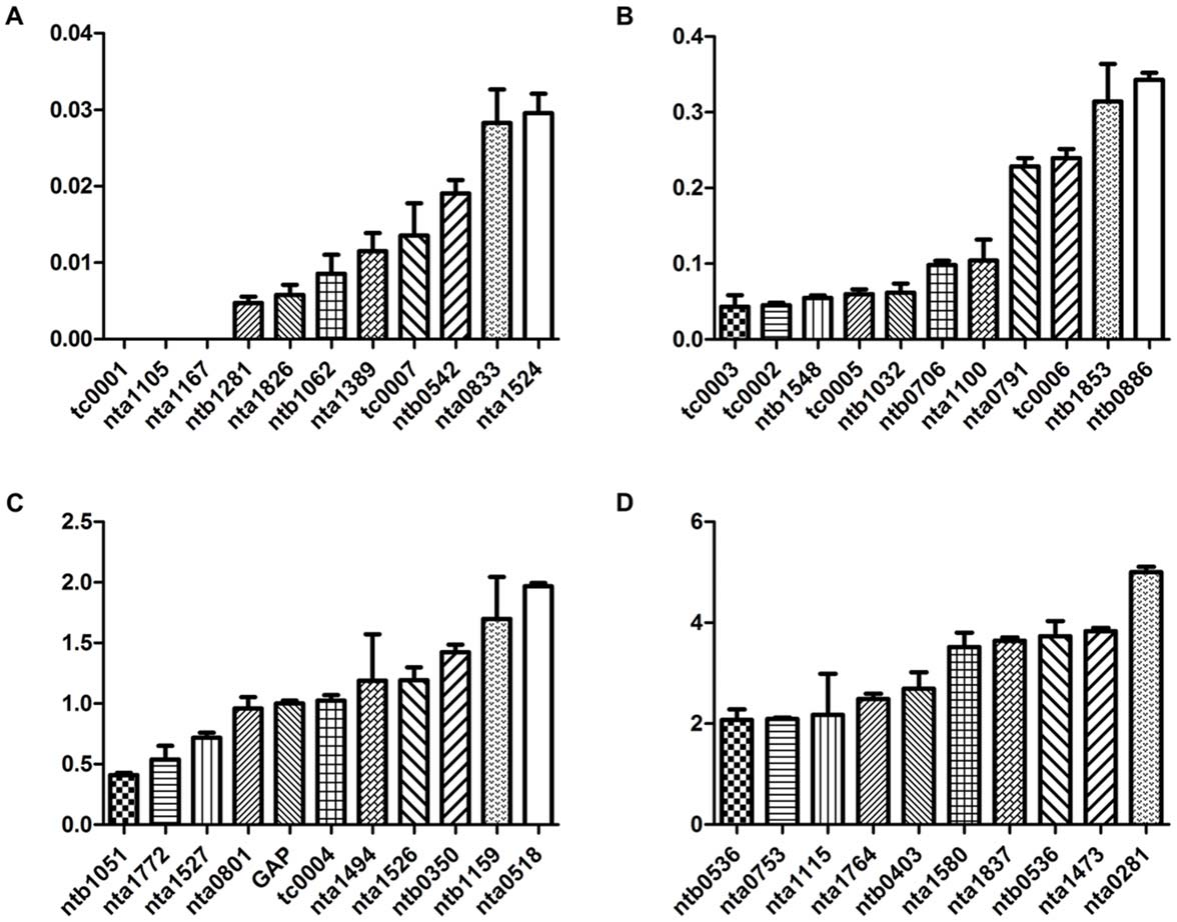
qPCR results showing expressed sequence tags (ESTs) existence in zygote cells. Of the 42 transcripts examined, 39 ESTs were found in zygote.

To estimate that the unevenly distributed transcripts are inherited from zygote or from *de novo* transcription in apical or basal cell, we also checked the 42 ESTs in zygote by qPCR. The result indicates that most of these transcripts were found in zygote and only three of them are not expressed in zygote (Figure 6; Table S2). This suggests most of the transcripts that preferentially distributed in apical or basal cell were inherited from zygote. Interestingly, among the three transcripts that not expressed in zygote, two (tc0001 and nta1105) specifically expressed in apical cell and not in basal cell (Figure 5), indicating cell-type specific *de novo* transcription occurred after zygotic division.

## Discussion

### Transcript diversity occurs between apical and basal cells after asymmetric zygote division

After zygotic cell division, the resulting apical and basal cells differ in terms of their morphology and developmental fate. Proper basal and apical cell formation is critical not only for embryo and suspensor differentiation, but also for the apical-basal axis establishment [15]–[17]. It has been unclear how two cells derived from the same mother cell can differentiate into distinct structures and how their fate is determined. In recent decades, different hypotheses, which are not mutually exclusive, were proposed to explain the distinct cell fates of this pair of sister cells. For example, cell fate could be determined by the position of the cells in the embryo sac, as the cell that is directly attached to the maternal tissue usually forms the suspensor [18]; cell fate could also be decided by an interaction between two attached or adjacent cells [19]–[23]. Another attractive hypothesis is that cytoplasmic determinants may play a predominant role in cell fate determination [7]. Asymmetric cell division generates daughter cells containing different developmental determinants [5]. In fact, some genes that may be involved in cell fate determination have been reported in *Arabidopsis*; for example, *WOX2* expression is confined to apical daughter cells of the zygote, while *WOX9* expression is initiated in the basal daughter cell of the zygote [9]. Our data confirm that the diversity of transcript profiles indeed occurs between apical and basal cells in tobacco. RT-PCR and qPCR further confirm the difference of transcript composition between the two cell types, suggesting that the diversity of transcript profiles in the cells is at least initiated at two-celled proembryo stage. However, it is difficult to calculate the percentage of differentially expressed transcripts in the whole transcript composition of apical or basal cells due to the limitation of transcript profile analysis (Table S3, S4). Further transcriptome analysis based on novel sequencing technique will enable a more complete survey of the diversity or similarity between apical and basal cells at the transcriptional level and a more accurate answer to this question.

### Preferential distribution of zygote transcripts and cell type specific *de novo* transcription may be involved in apical/basal cell fate determination

To explain the mechanism of basal cell and suspensor specification, scientists ever proposed that basal cell–specifying morphogenetic factors are distributed asymmetrically in the cytoplasm of the egg cell or zygote. Upon asymmetric division, these factors are inherited by the basal cell and trigger the transcription of basal region–specific genes and, eventually, the specification of suspensor [5].

In our experiments, we confirmed that among 42 ESTs examined by real-time RT-PCR, 19 showed significant expression level differences between apical and basal cells (Figure 5 and 6; Table S2). We further confirmed that the most of these transcripts were inherited from zygote. Thus, it is likely that these zygotic transcripts are preferentially portioned into the different daughter cells during asymmetric cell division. This finding offer direct evidence for Weterings' proposal.

Among thirteen transcripts expressed at significantly higher levels in apical cells than in basal cells, Tc0003 encodes part of the open reading frame, and it possess 20 N-terminal amino acids of the basic helix-loop-helix (bHLH) domain [24], [25]. BIM1, a bHLH protein involved in brassinosteroid signaling, contributes to embryo patterning via its interaction with AP2-type transcription factors [26]. Nta1473 is a P0-related acidic ribosomal protein. *ZmrpP0* is relatively abundantly expressed in unfertilized egg cells but down regulated in zygotes by 18 hour after *in-vitro* fertilization [27]. Nta1826 is similar to the *Arabidopsis* transcription factor NF-YB8 (NUCLEAR FACTOR Y, SUBUNIT B8). Of the ten *Arabidopsis* HAP3 subunits, NF-YB8 is a non-LEC1-type protein, while LEC1-type HAP3 subunits LEC1 and L1L define a class of regulators essential for embryo development [28]. Among four transcripts expressed at significantly higher levels in basal cells than in apical cells, tc0002 is similar to a Leu zipper domain containing transcription factor. A reported such transcription factor in *Arabidopsis*, GLUTAMINE-RICH PROTEIN23 (GRP23), is essential for early embryogenesis [29]. The *grp23* showed abnormal division in both apical and basal cell development. nta1115 and ntb1853 are quite similar to WOX9. Interestingly, nta1527 is quite similar to WOX2 and was expressed more highly in apical cells than in basal cells. In *Arabidopsis*, *WOX2* expression is confined to apical cells, whereas *WOX9* expression has been reported in basal cells [9]. These genes play critical role in early embryogenesis and embryo pattern formation.

We also confirmed that two ESTs (tc0001 and nta1105) existed exclusively in apical cells and not in zygotes, indicating that cell type specific *de novo* transcription occurs after zygotic cell division. Tc0001 showed no similarity to any sequence in the NCBI or TAIR database. nta1105 is similar to the *Arabidopsis* transcription factor HSFB2B (class B heat shock factor). HsfB2b is involved in the regulation of Pdf1.2 defensin gene expression and pathogen resistance in *Arabidopsis* [30]. The function of such *de novo* transcripts remain to be elucidated. Their occurrence only in apical cell may suggest their potential role in embryogenesis.

In summary, we conclude that zygotic division results in a divergence in apical and basal cell transcript profiles. The asymmetric zygotic division indeed brings unequal or uneven distribution of embryogenesis-related transcripts in apical and basal cell. We have further confirmed that these transcripts previously exist in zygote. The cell-type specific *de novo* transcription in these two cell types also occurs. Thus, both the preferential distribution of zygotic transcripts and the cell-type specific *de novo* transcription may contribute to the different composition of transcripts in apical or basal cells.

## Materials and Methods

### Apical and basal cell isolation from tobacco

Tobacco (*Nicotiana tabacum* cv SR1) plants were grown in a greenhouse at 25°C with a light period of 16 h. Two-celled proembryos were isolated by enzymatic maceration combined with brief manual grinding [13]. During the isolation process, two transcription inhibitors, actinomycin D (50 mg/L) and cordycepin (100 mg/L), were added to all solutions to inhibit potential stress-induced gene expression [12], [14].

An LMD (Leica, Bonn, Germany) was used to destroy one of the cells in two-celled proembryos to obtain the apical or basal cell, respectively. The ablation solution was 13% mannitol. To destroy the apical cell, the “move” mode was chosen to operate laser spot since two-celled proembryos was not fixed and might move on the film. A tailor-made slide with PET film at the bottom was used as a cell container during the ablation. A 5µl microdrop of mannitol solution was first transferred onto the film and then two-celled proembryos were transferred into the droplet and finally the slide was covered with a coverslip to avoid evaporation. The energy of laser spot was modulated (30–60% of the full power, 200W) frequently to realize ablation. The parameter of “specimen balance” was set at 2.

Isolated apical or basal cells were gently washed twice with 13% mannitol and transferred to lysis/binding buffer (Dynal Biotech, Oslo, Norway) for mRNA isolation. The viability of the isolated apical and basal cells was confirmed by FDA staining (Figure S1).

### cDNA library construction and sequencing

A Dynabeads mRNA DIRECT Micro Kit (Dynal Biotech) was used for mRNA isolation. A SMART cDNA Library Construction Kit (Clontech Laboratories, Mountain View, CA, USA) was used for cDNA library construction, following the manufacturer's instructions. Ligations were packaged with Gigapack III Gold packaging reagents (Stratagene, La Jolla, CA, USA). Individual cloned cDNAs were obtained by *in vivo* mass excision, randomly picked, and sequenced using a DNA capillary sequencer (ABI 3730XL, Applied Biosystems, Foster City, CA, USA).

### Bioinformatics

ESTs from the two individual libraries were combined for the following processes. PHRED was used for base calling and trimming of low-quality sequences. Cloning vectors and linkers were masked with the CROSS-MATCH program. The cleaned EST sequences were clustered using Uicluster2 and assembled into clusters using Cap3. Groups containing only one sequence were classified as singletons. For annotation, the assembled consensus sequences and singletons were used as a query for BLASTN and BLASTX searches (http://www.ncbi.nlm.nih.gov). The e-values were 10^−5^ for both BLASTN and BLASTX. Clusters encoding proteins of known function were categorized manually into broad functional groups according to the FunCat annotation scheme. For comparative analysis of ESTs derived from different cDNA libraries(apical cell, basal cell, zygote, two-celled proembryo), all these ESTs were clustered using Blastclust software. All the ESTs were submitted to NCBI database. The accession numbers for apical cell ESTs are HS080288–HS083059, for basal cell ESTs are HS083060–HS085835, and for two-celled proembryo ESTs (tc0001–tc0007) are HO844849, HO844115, HO845026, HO843422, HO844377, HO844521, and HO844358.

### RT-PCR expression analysis

mRNA was isolated from apical cells, basal cells, global-stage embryos, and heart-shaped embryos. First-strand cDNA was synthesized using Oligo (dT)15 (Sigma, Hamburg, Germany) and Superscript III Reverse Transcriptase (Invitrogen, Carlsbad, CA, USA); a Super SMART™ cDNA PCR Synthesis Kit (Clontech Laboratories) was used for cDNA amplification. The tobacco glyceraldehyde-3-phosphate dehydrogenase (GAPDH) gene (GenBank accession no. AJ133422, GAPs: 5′-TCCACTCCATCACAGCCACA-3′, GAPas: 5′-AGACTCCTCACAGCAGCACC-3′) was used as a control. RT-PCR was done as described previously [12].

### Real-time RT-PCR analysis

The quantitative expression of apical/basal cell-derived transcripts was estimated by real-time RT-PCR using single-stranded cDNA from specific cells preamplified using a Super SMART™ cDNA PCR Synthesis Kit. cDNAs from apical/basal cells were used as the template for real-time PCR, with gene-specific primers in 20-µl reactions containing 1× FastStart Universal SYBR Green Master (Roche Diagnostics, Mannheim, Germany), and 300 nM each primer. Real-time PCR was performed over 45 cycles (95°C for 15 s and 60°C for 1 min) with a Roter-Gene 6000 system (Corbett Research, Mortlake, Australia). The data were analyzed using LinRegPCR [31]. In pre-experiments, expression level of the six reference genes were tested in apical/basal cell and zygote: GAPDH(AJ133422), Actin(GQ281246), Polyubiquitin(GQ281244), L25 ribosomal protein(L18908), Elongation factor 1a (EF-1a; AF120093) and Ubiquitin-conjugating enzyme E2 (Ntubc2; AB026056). Stability of the reference genes was examined by geNORM v3.5 [32]. Finally, GAPDH/Polyubiquitin/Ntubc2 were chosen for the calculation of normalization factor. Relative expression level was then calculated based on the N0 in LinRegPCR and normalization factor in geNORM. Thus, for each examined gene, the expression levels of the examined gene in the apical/basal cell samples were normalized to the expression levels of reference genes, GAPDH/Polyubiquitin/Ntubc2. Primer pairs for reference genes are: qGAPs: 5′-AGGCTGGAGAAAGAAGCTACCTA-3′, qGAPas: 5′-AGTCTGTGGACACCACATCATCT-3′; qUbis: 5′-GCGGTGGTATGCAGATTTTC-3′, qUbias: 5′-TCCTGCAAAGATCAGCCTCT-3′; qUBC2s: 5′-CTGGACAGCAGACTGACATC-3′, qUBC2as: 5′-CAGGATAATTTGCTGTAACAGATTA-3′.

## Supporting Information

Figure S1 Viability of two-celled proembryo, isolated apical and basal cell.
(TIF)

Figure S2 Transcript levels of selected transcripts.
(TIF)

Table S1 Result of RT-PCR and detailed information of ESTs examined.
(XLS)

Table S2 Result of qPCR and detailed information of ESTs examined.
(XLS)

Table S3 Detailed information on apical cell ESTs.
(XLS)

Table S4 Detailed information on basal cell ESTs.
(XLS)

## References

[1] Gallagher K, Smith LG (1997). Asymmetric cell division and cell fate in plants.. Curr Opin Cell Biol.

[2] Scheres B, Benfey PN (1999). Asymmetric cell division in plants.. Annu Rev Plant Physiol Plant Mol Biol.

[3] He YC, He YQ, Qu LH, Sun MX, Yang HY (2007). Tobacco zygotic embryogenesis *in vitro*: the original cell wall of the zygote is essential for maintenance of cell polarity, the apical-basal axis and typical suspensor formation.. Plant J.

[4] Lindsey K, Topping JF (1993). Embryogenesis: a question of pattern.. J Exp Bot.

[5] Weterings K, Apuya NR, Bi Y, Fischer RL, Harada JJ (2001). Regional localization of suspensor mRNAs during early embryo development.. Plant Cell.

[6] Laux T, Wurschum T, Breuninger H (2004). Genetic regulation of embryonic pattern formation.. Plant Cell.

[7] Gurdon JB (1992). The generation of diversity and pattern in animal development.. Cell.

[8] Lu P, Porat R, Nadeau JA, O'Neill SD (1996). Identification of a meristem L1 layer-specific gene in *Arabidopsis* that is expressed during embryonic pattern formation and defines a new class of homeobox genes.. Plant Cell.

[9] Haecker A, Gross-Hardt R, Geiges B, Sarkar A, Breuninger H (2004). Expression dynamics of *WOX* genes mark cell fate decisions during early embryonic patterning in *Arabidopsis thaliana*.. Development.

[10] Xin HP, Sun MX (2010). What we have learned from transcript profile analyses of male and female gametes in flowering plants.. Chin Sci Bull.

[11] Okamoto T, Scholten S, Lorz H, Kranz E (2005). Identification of genes that are up- or down-regulated in the apical or basal cell of maize two-celled embryos and monitoring their expression during zygote development by a cell manipulation- and PCR-based approach.. Plant Cell Physiol.

[12] Ning J, Peng XB, Qu LH, Xin HP, Yan TT (2006). Differential gene expression in egg cells and zygotes suggests that the transcriptome is restructed before the first zygotic division in tobacco.. FEBS Lett.

[13] Fu CM, Sun MX, Zhou C, Yang HY (1996). Isolation of fertilized embryo sacs and zygotes and triggering of zygote division *in vitro* in *Nicotiana Tabacum*.. Acta Bot Sin.

[14] Leonhardt N, Kwak JM, Robert N, Waner D, Leonhardt G (2004). Microarray expression analyses of *Arabidopsis* guard cells and isolation of a recessive abscisic acid hypersensitive protein phosphatase 2C mutant.. Plant Cell.

[15] Jürgens G (2001). Apical-basal pattern formation in *Arabidopsis* embryogenesis.. EMBO J.

[16] Weijers D, Jurgens G (2005). Auxin and embryo axis formation: the ends in sight?. Curr Opin Plant Biol.

[17] De Smet I, Lau S, Mayer U, Jurgens G (2010). Embryogenesis - the humble beginnings of plant life.. Plant J.

[18] Priess JR, Schnabel H, Schnabel R (1987). The *glp-1* locus and cellular interactions in early *C. elegans* embryos.. Cell.

[19] van den Berg C, Willemsen V, Hage W, Weisbeek P, Scheres B (1995). Cell fate in the *Arabidopsis* root meristem determined by directional signaling.. Nature.

[20] van den Berg C, Willemsen V, Hendriks G, Weisbeek P, Scheres B (1997). Short-range control of cell differentiation in the *Arabidopsis* root meristem.. Nature.

[21] Vernon DM, Meinke DW (1994). Embryogenic transformation of the suspensor in *twin*, a polyembryonic mutant of *Arabidopsis*.. Dev Biol.

[22] Bouget F, Berger F, Brownlee C (1998). Position dependent control of cell fate in the *Fucus* embryo: role of intercellular communication.. Development.

[23] Irish VF, Jenik PD (2001). Cell lineage, cell signaling and the control of plant morphogenesis.. Curr Opin Genet Dev.

[24] Heim MA, Jakoby M, Werber M, Martin C, Weisshaar B (2003). The basic helix-loop-helix transcription factor family in plants: a genome-wide study of protein structure and functional diversity.. Mol Biol Evol.

[25] Toledo-Ortiz G, Huq E, Quail PH (2003). The *Arabidopsis* basic/helix-loop-helix transcription factor family.. Plant Cell.

[26] Chandler J, Cole M, Flier A, Werr W (2009). BIM1, a bHLH protein involved in brassinosteroid signaling, controls *Arabidopsis* embryonic patterning via interaction with DORNRÖSCHEN and DORNRÖSCHEN-LIKE.. Plant Mol Biol.

[27] Dresselhaus T, Cordts S, Heuer S, Sauter M, Lörz H (1999). Novel ribosomal genes from maize are differentially expressed in the zygotic and somatic cell cycles.. Molecular and General Genetics MGG.

[28] Kwong RW, Bui AQ, Lee H, Kwong LW, Fischer RL (2003). LEAFY COTYLEDON1-LIKE defines a class of regulators essential for embryo development.. Plant Cell.

[29] Ding YH, Liu NY, Tang ZS, Liu J, Yang WC (2006). *Arabidopsis GLUTAMINE-RICH PROTEIN23* is essential for early embryogenesis and encodes a novel nuclear PPR motif protein that interacts with RNA polymerase II subunit III.. Plant Cell.

[30] Kumar M, Busch W, Birke H, Kemmerling B, Nurnberger T (2009). Heat shock factors HsfB1 and HsfB2b are involved in the regulation of Pdf1.2 expression and pathogen resistance in *Arabidopsis*.. Mol Plant.

[31] Ramakers C, Ruijter JM, Deprez RH, Moorman AF (2003). Assumption-free analysis of quantitative real-time polymerase chain reaction (PCR) data.. Neurosci Lett.

[32] Vandesompele J, De Preter K, Pattyn F, Poppe B, Van Roy N (2002). Accurate normalization of real-time quantitative RT-PCR data by geometric averaging of multiple internal control genes.. Genome Biol.

