# Supplementary figures and images for: Transcription Profile Analysis Reveals That Zygotic Division Results in Uneven Distribution of Specific Transcripts in Apical/Basal Cells of Tobacco
Source: PLoS One. 2011 Jan 7;6(1):e15971. doi: 10.1371/journal.pone.0015971 (PMC3017550; doi:10.1371/journal.pone.0015971)

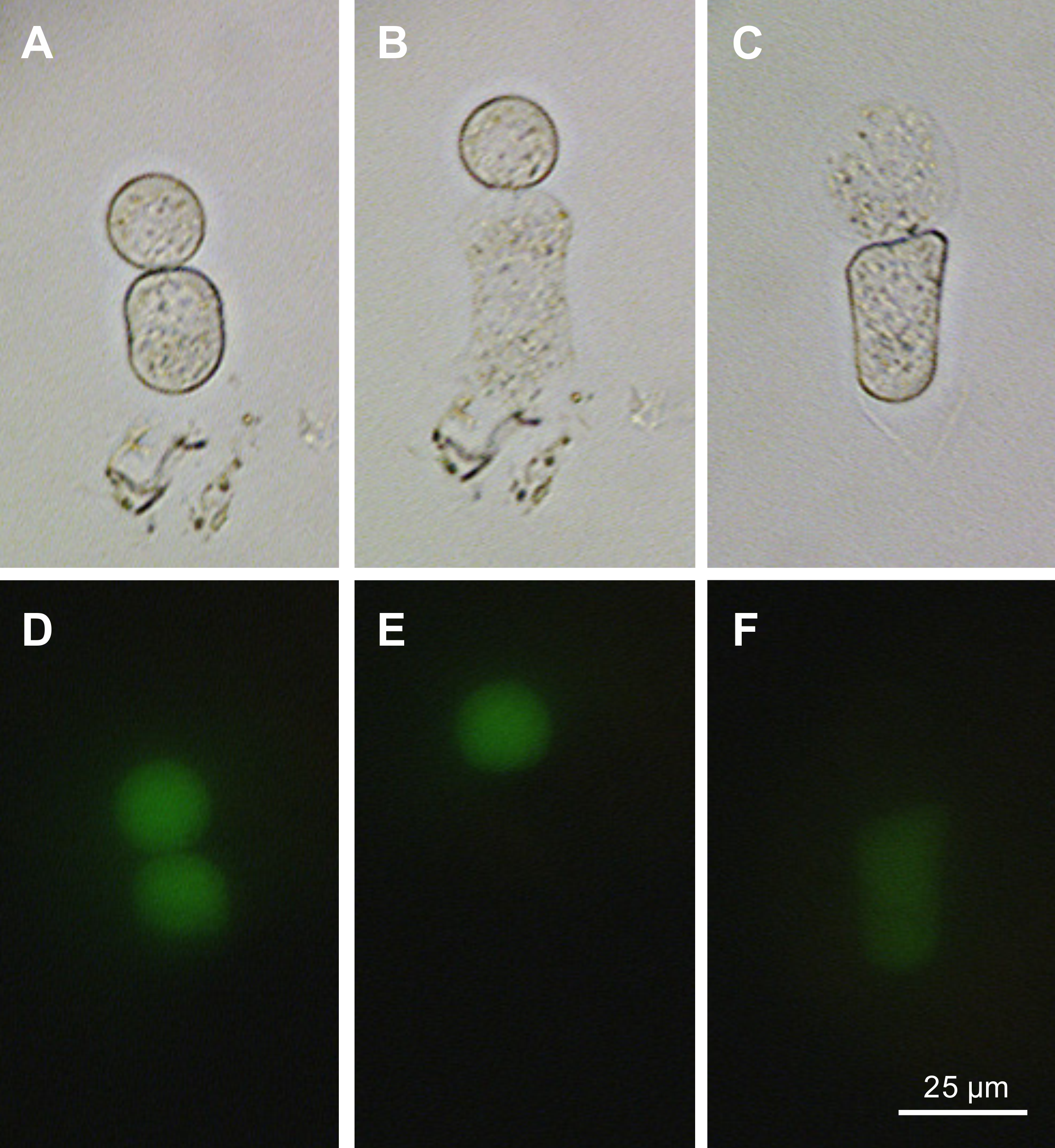

Supplement: Figure S1 — Viability of two-celled proembryo, isolated apical and basal cell. (TIF) [file pone.0015971.s001.tif]

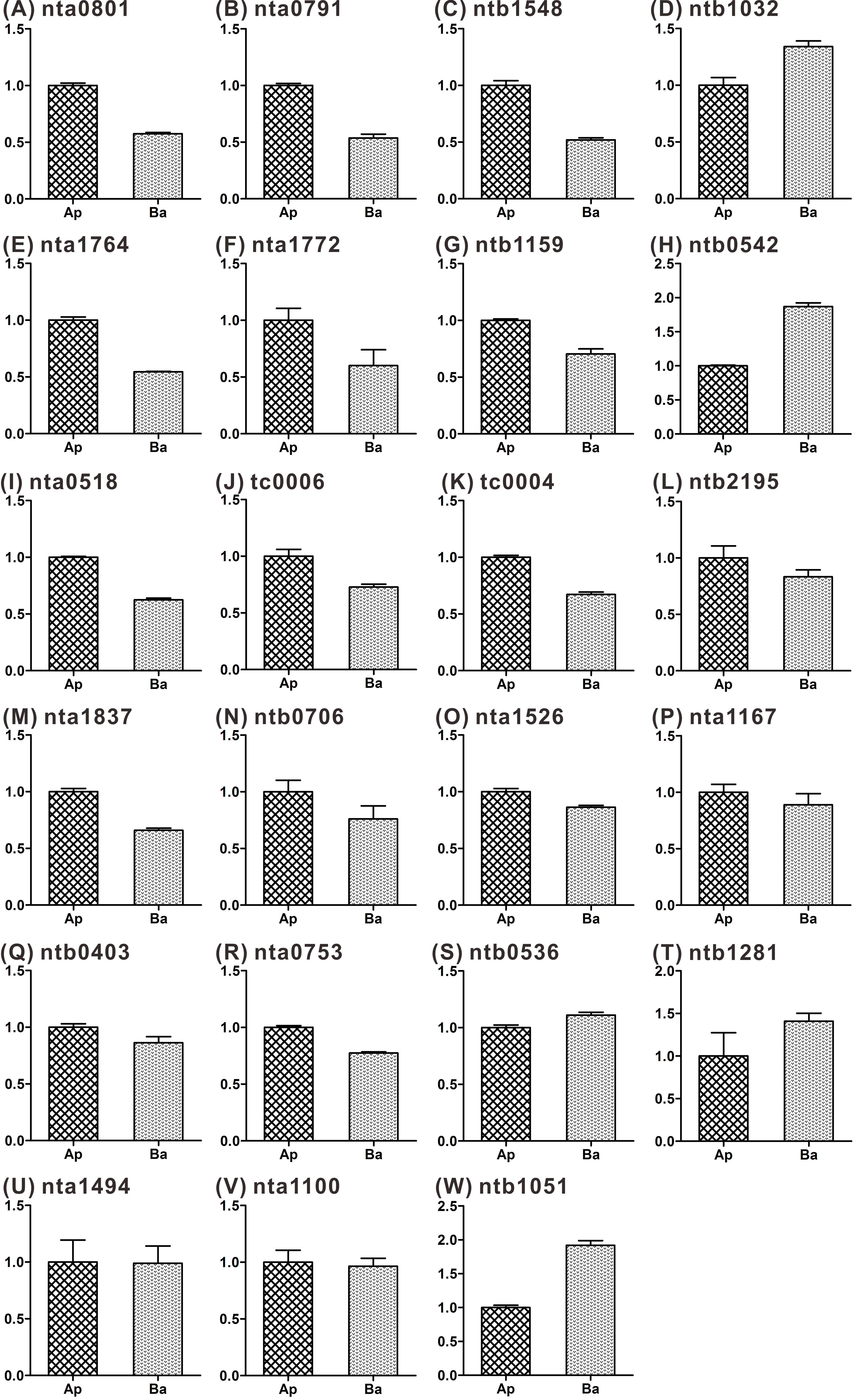

Supplement: Figure S2 — Transcript levels of selected transcripts. (TIF) [file pone.0015971.s002.tif]
